# The Effects of Upper-Limb Training Assisted with an Electromyography-Driven Neuromuscular Electrical Stimulation Robotic Hand on Chronic Stroke

**DOI:** 10.3389/fneur.2017.00679

**Published:** 2017-12-14

**Authors:** Chingyi Nam, Wei Rong, Waiming Li, Yunong Xie, Xiaoling Hu, Yongping Zheng

**Affiliations:** ^1^Department of Biomedical Engineering, The Hong Kong Polytechnic University, Hong Kong

**Keywords:** stroke, hand, rehabilitation, robot, neuromuscular electrical stimulation

## Abstract

**Background:**

Impaired hand dexterity is a major disability of the upper limb after stroke. An electromyography (EMG)-driven neuromuscular electrical stimulation (NMES) robotic hand was designed previously, whereas its rehabilitation effects were not investigated.

**Objectives:**

This study aims to investigate the rehabilitation effectiveness of the EMG-driven NMES-robotic hand-assisted upper-limb training on persons with chronic stroke.

**Method:**

A clinical trial with single-group design was conducted on chronic stroke participants (*n* = 15) who received 20 sessions of EMG-driven NMES-robotic hand-assisted upper-limb training. The training effects were evaluated by pretraining, posttraining, and 3-month follow-up assessments with the clinical scores of the Fugl-Meyer Assessment (FMA), the Action Research Arm Test (ARAT), the Wolf Motor Function Test, the Motor Functional Independence Measure, and the Modified Ashworth Scale (MAS). Improvements in the muscle coordination across the sessions were investigated by EMG parameters, including EMG activation level and Co-contraction Indexes (CIs) of the target muscles in the upper limb.

**Results:**

Significant improvements in the FMA shoulder/elbow and wrist/hand scores (*P* < 0.05), the ARAT (*P* < 0.05), and in the MAS (*P* < 0.05) were observed after the training and sustained 3 months later. The EMG parameters indicated a significant decrease of the muscle activation level in flexor digitorum (FD) and biceps brachii (*P* < 0.05), as well as a significant reduction of CIs in the muscle pairs of FD and triceps brachii and biceps brachii and triceps brachii (*P* < 0.05).

**Conclusion:**

The upper-limb training integrated with the assistance from the EMG-driven NMES-robotic hand is effective for the improvements of the voluntary motor functions and the muscle coordination in the proximal and distal joints. Furthermore, the motor improvement after the training could be maintained till 3 months later.

**Trial registration:**

ClinicalTrials.gov. NCT02117089; date of registration: April 10, 2014.

## Introduction

Stroke is one of the leading causes of adult disability, and patients with stroke require continuous long-term medical care for reducing physical impairments (1). Only 18% of stroke survivors with severe paralysis achieve complete upper-limb function recovery within the subacute period (i.e., within the first 6 months after stroke onset) (2). Furthermore, approximately 65% of patients with chronic stroke (i.e., 6 months after the onset of a stroke) cannot incorporate their affected hand into their usual activities (3), this limitation markedly affects their independence and ability to perform activities of daily living (ADLs).

According to the traditional viewpoint on neurorehabilitation after stroke, significant motor improvements are usually observed during the subacute period and are associated with a spontaneous recovery in the early period, and motor recovery is expected to be minimal or plateaued during the chronic period (4). However, more recent studies on poststroke rehabilitation have reported that repetitive and high-intensity practice accelerate motor recovery (5, 6) and intensive therapeutic interventions can contribute significantly to reducing motor impairment and improving functional use of the affected arm of patients with chronic stroke (7). Despite these findings, providing high-intensity and repetitive training through traditional “one-to-one” manual-physical therapy is difficult because of resource constraints (8). Rehabilitation robots fill this gap by performing repetitive therapeutic tasks intensively and require minimal supervision by a therapist (9). Various robotic systems have been proposed for hand rehabilitation after stroke, for example, HapticKnob (10) and Haptic Master (11, 12), and their training effects had been investigated. These studies have reported that robot-assisted therapy can facilitate hand function recovery because the robotic system can provide repetitive and intensive training through a consistent and precise manner over a long duration. In addition, the integration of voluntary effort into robotic design for chronic stroke rehabilitation has been recommended (13, 14). Training designs that included this “add-on” feature of voluntary effort from the residual neuromuscular pathways exhibited better motor outcomes and longer sustainability than did passive limb motion training. Electromyography (EMG)-driven strategy is one of the novel and rapidly expanding techniques for maximizing the involvement of voluntary efforts during poststroke training. Many EMG-driven rehabilitation devices have been developed in the past decade (15), and a set of EMG-driven robot-assisted training systems have also been developed for poststroke rehabilitation in our previous studies (14, 16–18). The EMG-driven training systems have exhibited significant motor recovery for patients with chronic stroke, particularly in voluntary motor functions of the upper limb (15, 19).

While the EMG-driven strategy has been widely adopted, the use of robot-assisted therapy remains suboptimal. For example, a robot cannot directly activate the desired muscle groups because the target muscles of patients with stroke usually cooperate with compensatory motions from other muscular activities (20). In contrast to robot systems, neuromuscular electrical stimulation (NMES) can stimulate the required muscles by using electrical currents. NMES generates limb movement by precisely activating the target muscles to restore motor function and evoke sensory feedback to the brain during muscle contraction, thus promoting motor relearning (21). However, using NMES alone also has limitations in activating groups of muscles for dynamic limb movements. Controlling the speed of contraction of individual muscles during limb movements with desired kinematic qualities, such as speed, trajectory, and motion smoothness, is difficult mainly because of delayed evoked muscle contractions during electrical stimulation (22). Consequently, a combination of NMES and robot-assisted therapy has been developed recently for poststroke upper-limb rehabilitation (23–26), and studies have shown that this combination is effective for motor function recovery in patients with chronic stroke. In addition, the combined system can reduce the compensatory muscular activities at the elbow and can improve muscle activation levels related to the wrist in patients with chronic stroke, which was not observed when robot-assisted therapy was used alone (23). A study on the wrist training in patients with chronic stroke by another research group also demonstrated higher rehabilitation effectiveness in the upper-limb motor recovery with the combination therapy than with robot-assisted therapy alone (24).

Nevertheless, previous studies on combinations of NMES and robotic systems have mainly focused on motor recovery of the elbow and wrist joints (23–26). Thus far, only a few studies have reported EMG-driven NMES robot-assisted therapy for hand function recovery although loss of hand function is the primary factor of the upper-limb disability after stroke (27). In our previous work, an EMG-driven NMES exoskeletal hand robot, which could provide fine control of the hand movements and activating the target muscles selectively for fingers extension/flexion, was developed and suggested for hand rehabilitation after stroke (28), where the assistive capacity of the NMES and robot combined system in helping persons with chronic stroke conducting extension/flexion of the fingers were compared with either NMES or robot assistive schemes. NMES and robot combined scheme showed higher motion accuracy and better muscle coordination in the whole upper limb. However, the rehabilitation efficacy and the training effects had not been well investigated by clinical trials previously. In this work, the rehabilitation effectiveness of the EMG-driven NMES-robotic hand assisted upper-limb training on chronic stroke was investigated by a single-group trial. We hypothesized that the participants who received intensive and repetitive upper-limb training with coordinated hand movements assisted by the EMG-driven NMES-robotic hand would demonstrate improvements in hand function and muscular coordination of the fingers. Furthermore, motor gains after the training could contribute to the functional use of the affected hand in ADLs.

## Methodology

### EMG-Driven NMES-Robotic Hand

**Figure 1:**
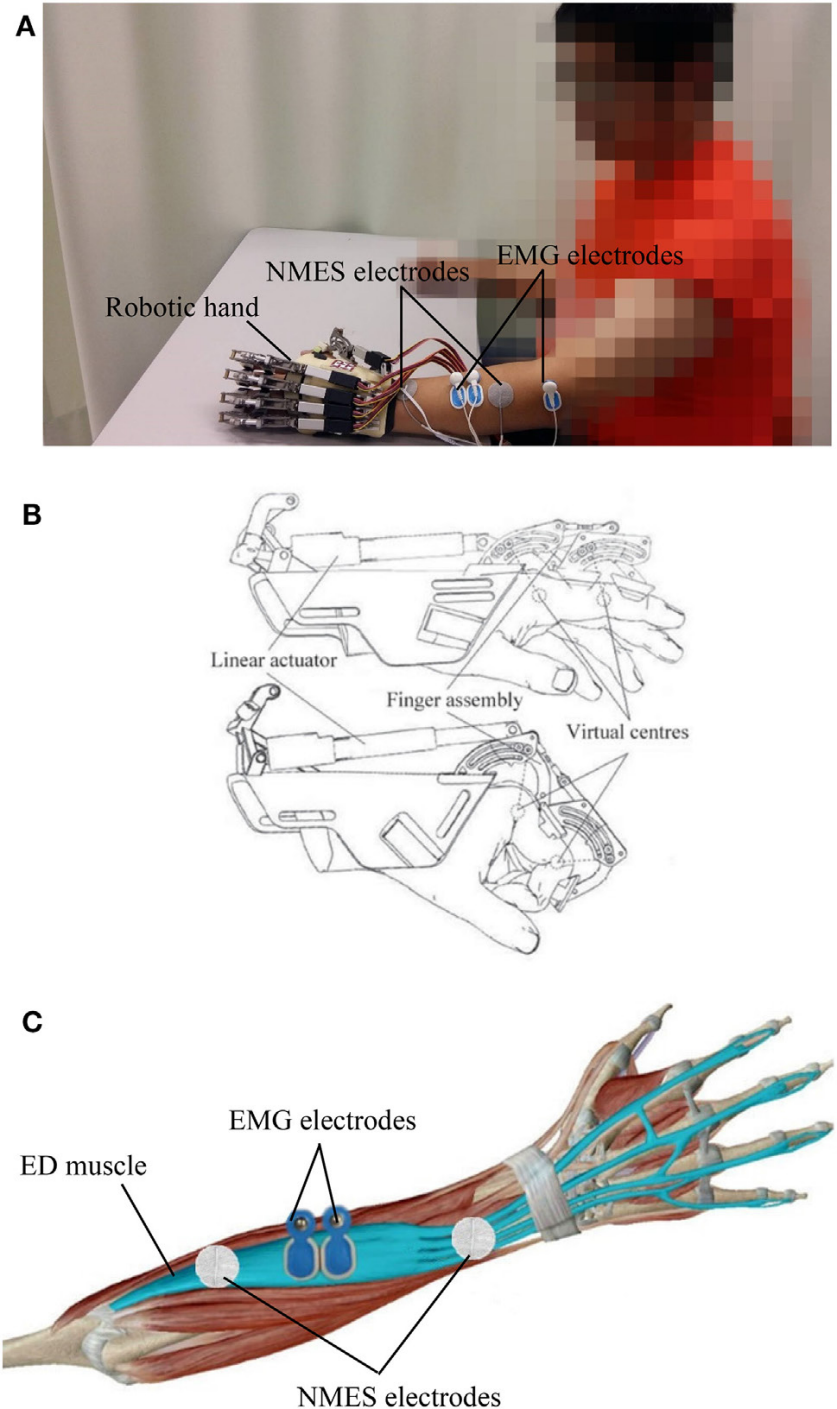
The electromyography (EMG)-driven neuromuscular electrical stimulation (NMES)-robotic hand system: **(A)** the wearable system consisting of a mechanical exoskeleton of the robotic hand, a pair of NMES electrodes attached to the extensor digitorum (ED) muscle, and EMG electrodes on the ED and abductor pollicis brevis muscles; **(B)** illustration of the mechanical structure of the robotic hand; **(C)** the EMG and NMES electrodes’ configuration on the ED muscle.

The EMG-driven NMES-robotic hand system used in this study is shown in Figure 1A. The system can provide assistance for finger extension and flexion of the paretic limb of patients with stroke.

The wearable robotic hand (Firgelli L12, Firgelli Technologies Inc.) provided individual mechanical assistance to the five fingers, and each finger was actuated by a linear actuator (Figure 1B) (28, 29). The proximal and distal section of the index, middle, ring, and little fingers were rotated around the virtual centers located at the metacarpophalangeal (MCP) and proximal interphalangeal (PIP). The thumb was rotated around the virtual center of its MCP joint. The finger assembly provided two degrees of freedom for each finger and offered a range of motion (ROM) of 55° and 65° for the MCP and PIP joints, respectively. The angular rotation speeds of the two joints were set as 22°/s and 26°/s at the MCP and PIP joints, respectively, during training.

The NMES electrode pair (30 mm diameter; Axelgaard Corp., Fallbrook, CA, USA), which provided stimulation during finger extension, was attached over the extensor digitorum (ED) muscle. The configuration for the EMG and NMES electrodes on the ED muscle is shown in Figure 1C. The outputs of NMES were square pulses with a constant amplitude of 70 V, stimulation frequency of 40 Hz, and a manually adjustable pulse width in the range 0–300 µs. Before the training, the pulse width was set at the minimum intensity, which achieved a fully extended position of the fingers in each patient. No assistance from NMES was provided during finger flexion to avoid the possible increase of finger spasticity after stimulation (30).

The abductor pollicis brevis (APB) and ED muscles were used as voluntary neuromuscular drives to control robot assistance and NMES assistance from the system to facilitate performance of phasic and sequential limb tasks, namely, hand closing and hand opening. The APB was selected as the driving muscle in the hand closing phase, since the EMG signals from the APB of the paretic limb after stroke are less affected by spasticity and are relatively easier to be controlled than the flexor digitorum (FD) muscle for finger movements in chronic stroke (31). EMG-triggered control was used in this study. Three times of the standard deviation (SD) above the EMG baseline in the resting state was set as a threshold level in each motion phase during training. In the “hand closing” phase, as soon as the EMG activation level of the APB muscle reached a preset threshold (3 SD above the baseline), the robotic hand would close with a constant speed (22°/s and 26°/s at the MCP and PIP joints, respectively) and provide mechanical assistance for finger flexion motions. In the “hand opening” phase, once the EMG activation level of the ED muscle reached a preset threshold (3 SD above the baseline), the robotic hand would open with a constant speed (22°/s and 26°/s at the MCP and PIP joints, respectively), and NMES would stimulate the ED muscle during the entire hand opening phase to assist finger extension motions. Once the assistance of the system was initiated, voluntary effort from the patient was not required and the assistance from the NMES and robotic parts would be continuously provided during the entire hand closing and opening phase in the defined ROM.

The EMG signals from the driving muscles captured using EMG electrodes (Blue Sensor N, Ambu Inc. with a contact area of 20 mm × 30 mm) were first amplified 1,000 times (preamplifier: INA 333; Texas Instruments Inc., Dallas, TX, USA), sampled at 1,000 Hz by using a data acquisition card (DAQ, 6218 NI DAQ card; National Instruments Corp.) and filtered by using a band-pass filter in the range 10–500 Hz. After digitization, the EMG signals from the APB and ED muscles were rectified and low-pass filtered (fourth-order, zero-phase forward and reverse Butterworth filter; cutoff frequency, 10 Hz) to obtain an envelope of EMG signals (i.e., the EMG activation level) in the real-time control.

### Participants

**Figure 2:**
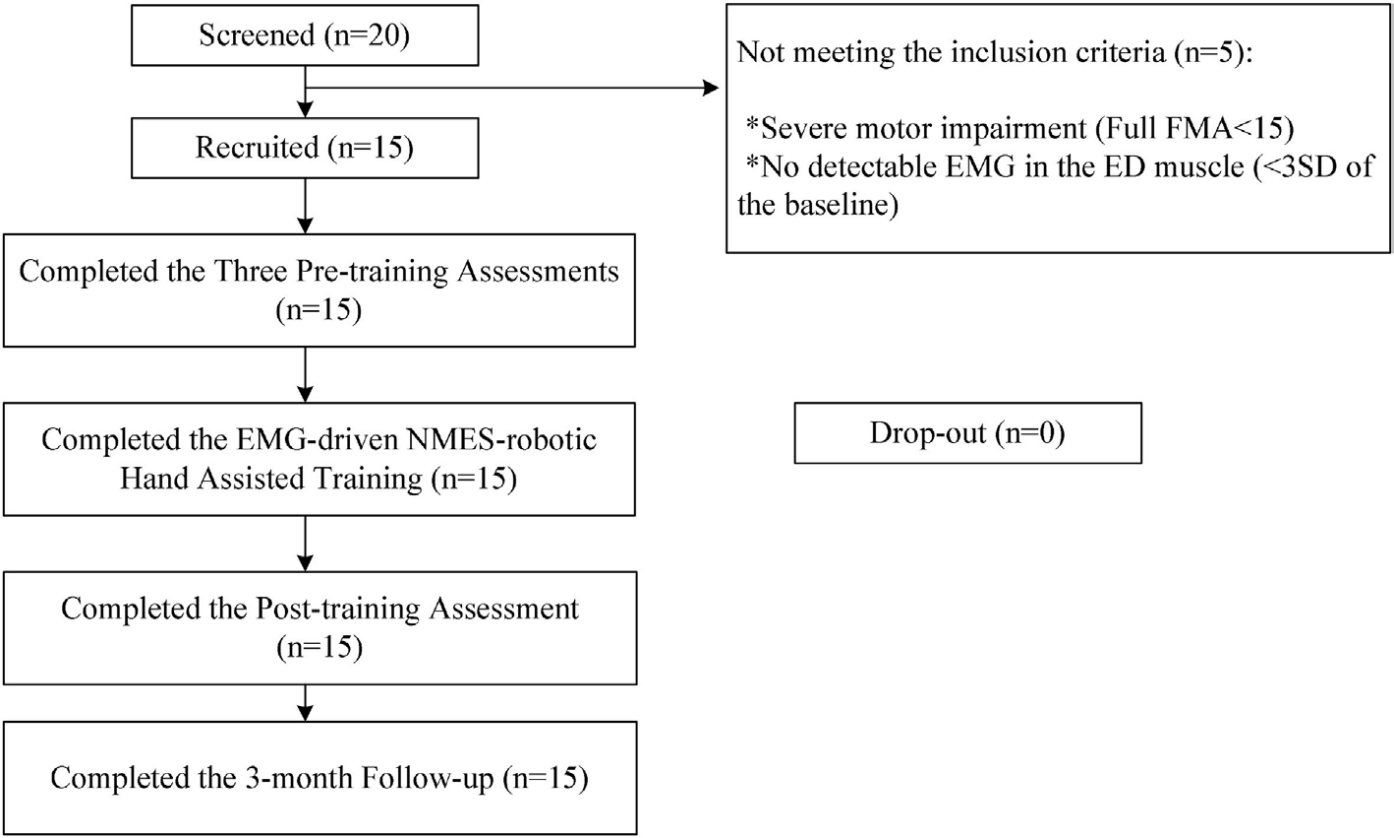
The Consolidated Standards of Reporting Trials flowchart of the experimental design.

After obtaining ethical approval from the Human Subjects Ethics Subcommittee of the Hong Kong Polytechnic University, participants in this study were recruited from the local districts through advertisement. A total of 20 patients were screened for the training during the subject recruitment. Fifteen participants with chronic poststroke hemiparesis met the inclusion criteria and were recruited in this study after obtaining their written consents. Inclusion criteria were as follows: (1) aged from 18 to 78 years (32, 33), (2) at least 6 months after the onset of a singular and unilateral brain lesion due to stroke, (3) both the MCP and PIP joints could be extended to 180° passively, (4) the spasticity during extension at the finger joints and the wrist joint was below 3 as measured by the Modified Ashworth Scale (MAS), ranged from 0 (no increase in the muscle tone) to 4 (affected part rigid) (34), (5) motor impairments in the affected upper limb ranged from severe to moderate as assessed by Fugl-Meyer Assessment (FMA) (15 < FMA < 45, with a maximal score of 66 for the upper limb) (35), (6) no visual deficit and able to understand and follow simple instructions as assessed by the Mini-Mental State Examination (MMSE > 21) (36), (7) detectable voluntary EMG signals from the driving muscle on the affected side (three times of the SD above the EMG baseline). Subjects were excluded because of the following conditions: (1) did not fulfill the above inclusion criteria, (2) currently pregnant, and (3) had an implanted pacemaker. Figure 2 shows the CONSORT flowchart of the experimental design.

### Training Protocol

All participants received the EMG-driven NMES-robotic hand-assisted upper-limb training, which consisted of 20 training sessions with the intensity of 3–5 sessions/week, within 7 consecutive weeks.

#### Session-by-Session Pretraining Evaluation Task

An evaluation was conducted at the beginning of each training session. Each participant was first subjected to a maximum voluntary contraction (MVC) test for the following five target muscles: APB, ED, FD, biceps brachii (BIC), and triceps brachii (TRI). EMG electrode pairs with center separation of 2 cm were attached to the skin surface of the muscles of interest according to the configuration specified in Cram’s work (37). Then, each participant was instructed to perform a bare hand evaluation task, which was used to monitor the muscle coordination during the recovery, as we did previously in EMG-driven hand robot-assisted upper-limb training of patients with chronic stroke (29). During evaluation, participants were seated at a table to maintain a vertical distance of 30–40 cm between the table surface and the participants’ shoulder.

While conducting the MVC test on the ED and FD muscles, participants were seated at a table and the paretic upper limb was placed on the table with elbow joint extended at an angle of 130°, the wrist was held by an experimental operator and positioned around its neutral position, and the finger positions were set by the operator to obtain an angle around 150° at the MCP joints of the index, middle, ring, and little fingers. During the maximum extension of the four fingers, the ED EMG signals were recorded; and during the maximum flexion, the FD EMG signals were captured. For the MVC test on the APB, the operator held the thumb in an extended position (around 30°) and asked the participants to conduct maximum thumb palmar abduction with the same configuration of the wrist and elbow joints as in the ED and FD MVC tests. During the MVC test on the BIC and TRI muscles, the paretic upper limb was positioned with the shoulder abducted at 70° and the elbow flexed at 90°. The MVC test on each target muscle was repeated twice and was maintained for 5 s. A 2-min interval was maintained between two consecutive contractions to prevent muscle fatigue. The maximum EMG amplitude recorded in the two repetitions was selected as the EMG amplitude of MVC for the target muscle.

The bare hand evaluation task, which was conducted after the MVC test, involved lateral and vertical arm reaching-grasping tasks (29). The participants were required to use their paretic limbs to perform the task (without assistance from NMES or the robotic hand) and complete it at their natural speed. In the lateral task, each participant was instructed to grasp a sponge (thickness 5 cm, weight 30 g) that was placed on one side of a table near the paretic side of the participant, transport the sponge 50 cm horizontally, release it, grasp it again, and return it to the starting point. In the vertical task, each participant was instructed to grasp the sponge on the midline of the lower layer of a shelf, lift it through a vertical distance of 17 cm, place it on the midline of the upper layer of the shelf, grasp it again, and place it back on the starting point. Both lateral and vertical tasks were repeated thrice, with a 2-min interval between two consecutive contractions to prevent muscle fatigue.

The EMG recording was started when the participant began to grasp the sponge (as soon as one finger touched the sponge) to when the participant released the sponge at the starting point (all the fingers left the sponge). The EMG signals from the target muscles (APB, ED, FD, BIC, and TRI) were first amplified 1,000 times, filtered by a band-pass filter in the range 10–500 Hz, and full-wave rectified. The EMG signals were sampled at 1,000 Hz by the data acquisition card and stored in the computer for off-line processing as we did previously (16, 17). In the early sessions of the training, only two participants could release the sponge without using their unaffected hands. A 10-s maximum time limit was set at the end of the attempt of release action for participants who could not release the sponge within 10-s by using their paretic hands. If their paretic hands could not release the sponge within 10-s, the participants could use their unaffected hands to remove the sponge. The EMG signals beyond 10-s were excluded for analysis. At the 20th session of training, five participants could release the sponge without using their unaffected hands.

#### Training Task Assisted with the EMG-Driven NMES-Robotic Hand

Participants were required to perform lateral and vertical arm reaching–grasping tasks with the EMG-driven NMES-robotic hand on the paretic side with same seating arrangement and movements as the previous evaluation. In each training session, the participants performed 30-min lateral and vertical tasks, respectively, with a 10-min interval between the tasks to prevent muscle fatigue. However, most of the participants (*n* = 12) could not sustain the weight of the paretic limb and the robotic hand without assistance. This was mainly due to weakness of the shoulder and elbow joints. Therefore, during the arm transportation, these participants were allowed to use their unaffected limb to provide self-aware minimal support at the wrist joint of the paretic limb. During the last session of the training, 10 participants could lift the affected limb while wearing the robotic hand.

### Evaluation of Training Effects

#### Clinical Assessments

In this study, the clinical assessments were used for functional evaluation of each participant and are described as follows: the FMA (35) that the full score is 66 for the upper-limb assessment and has been subscaled into shoulder/elbow (42/66) and wrist/hand (24/66) (38), used for poststroke measurement of motor functional impairment in voluntary limb movements; the Action Research Arm Test (ARAT) (39), adopted to evaluate the upper-limb functions with hand tasks included holding/releasing objects with different shapes, sizes and weights; the Wolf Motor Function Test (WMFT) (40), applied to collect the information on the motion speed and functional ability related to different daily tasks; the Motor Functional Independence Measure (FIM) (41), used for evaluation of subject’s ADLs; and the MAS (34), adopted to measure the spasticity of the flexors related to the elbow, wrist, and fingers. Before the training, the aforementioned clinical assessments were measured thrice in 2 weeks every 2–3 days to obtain the stability of baseline. The same clinical assessments were also measured immediately after the last training session and 3 months after the training by a training-blinded assessor who was instructed not to communicate regarding the training details with the participants and was not informed about the research purpose and the training protocol of this study.

#### EMG Parameters

**Figure 3:**
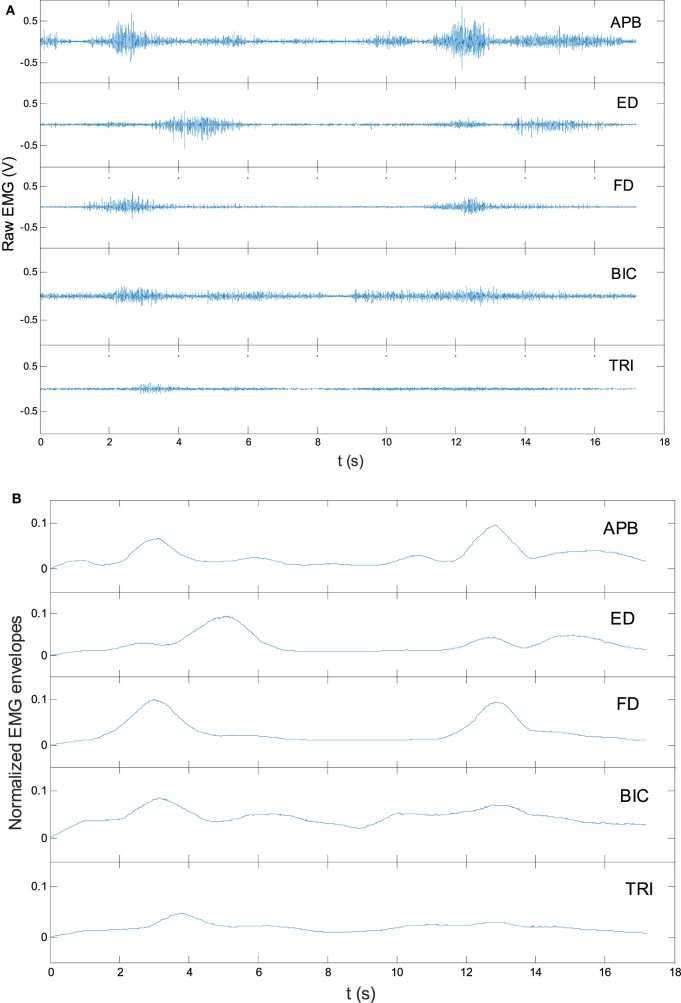
The representative raw electromyography (EMG) trials in a lateral arm reaching–grasping task **(A)** and the EMG envelopes after rectification and normalization **(B)**.

For the cross-sessional monitoring, two EMG parameters were calculated which were (1) the normalized EMG activation level of each target muscle and (2) the normalized EMG Co-contraction Index (CI) between a muscle pairs (16, 17). The EMG activation level of a muscle was calculated as follows:

(1) $$\bar{\text{EMG}}=\frac{1}{T}\int_{0}^{T}{\text{EMG}}_{i}(t)dt,$$

where $\bar{\text{EMG}}$ referred to the averaged EMG envelope value of muscle *i*. The EMG*_i_*(*t*) was the EMG envelope signal after the normalization with respect to the EMG MVC value of the muscle, and *T* was the length of the signal. Figure 3 shows the representative EMG signals, and their normalized envelopes captured during a trial of lateral reaching–grasping task. To minimize the variations in the EMG levels of individual participant, the obtained EMG activation level in a session for an individual participant was further normalized using the following equation (Eq. 2), which consider the maximal and minimal EMG activation levels of a participant recorded across the 20 training sessions. The tendency of the EMG activation level (values varying from 0 to 1) of a participant across the 20 training sessions was obtained after this operation.

(2) $${\text{EMG}}_{\text{N}}=\frac{\bar{\text{EMG}}-{\bar{\text{EMG}}}_{\text{min}}}{{\bar{\text{EMG}}}_{\text{max}-}{\bar{\text{EMG}}}_{\text{min}}},$$

where EMG_N_ was the normalized EMG activation level of muscle *i*. The $\bar{\text{EMG}}$ referred to the averaged EMG envelope value of muscle *i*. The ${\bar{\text{EMG}}}_{\text{min}}$ was the minimum value of the averaged EMG envelope across the 20 training sessions, and the ${\bar{\text{EMG}}}_{\text{max}}$ was the maximum value of the averaged EMG envelope across the 20 training sessions.

The CI between a pair of muscles were introduced and applied in our previous study and expressed as follows:

(3) $$\text{CI}=\frac{1}{T}\int_{0}^{T}A_{ij}(t)dt,$$

where *A_ij_*(*t*) was overlapping activity of EMG linear envelopes for muscles *i* and *j*, and *T* was the length of the signal. An increase in CI value represents an increased co-contraction phase of a muscle pair (broadened overlapping area), and a decrease in CI value indicates a decreased co-contraction phase of a muscle pair (lessened overlapping area). The CI value was also further normalized, similar to the EMG activation level, for obtaining the tendency of muscle coordination, which considers the maximal and minimal CI values of a participant recorded across the 20 training sessions and its equation (Eq. 4) was given as follows:

(4) $${\text{CI}}_{\text{N}}=\frac{\text{CI}-{\text{CI}}_{\text{min}}}{{\text{CI}}_{\text{max}}-{\text{CI}}_{\text{min}}},$$

where CI_N_ was the normalized CI value between a pair of muscles *i* and *j*. CI_min_ was the minimum value of the averaged overlapping activity of EMG linear envelopes, and CI_max_ was the maximum value of the averaged overlapping activity of EMG linear envelopes across the 20 training sessions. Session-by-session recording of the varying patterns of the two EMG parameters provided information particularly relevant to muscle activation and muscle coordination. Furthermore, it provided quantitative descriptions of the progress of motor function recovery of the affected limb.

### Statistical Analysis

The normality tests on the clinical scores and the EMG data by Lilliefors method were performed with a significant level of 0.05 (42). It found that the clinical score and the EMG sample had normal distribution (*P* < 0.05). One-way analysis of variance (ANOVA) with repeated measures (Bonferroni *post hoc* test) were used to evaluate the differences on the clinical assessments across different time points (thrice pretraining assessments, a posttraining assessment, and a 3-month follow-up assessment) and the EMG parameters (i.e., the normalized EMG activation levels and the CIs) across the 20 training sessions. The levels of statistical significance were indicated at 0.05, 0.01, and 0.001 in this study.

## Results

**Figure 4:**
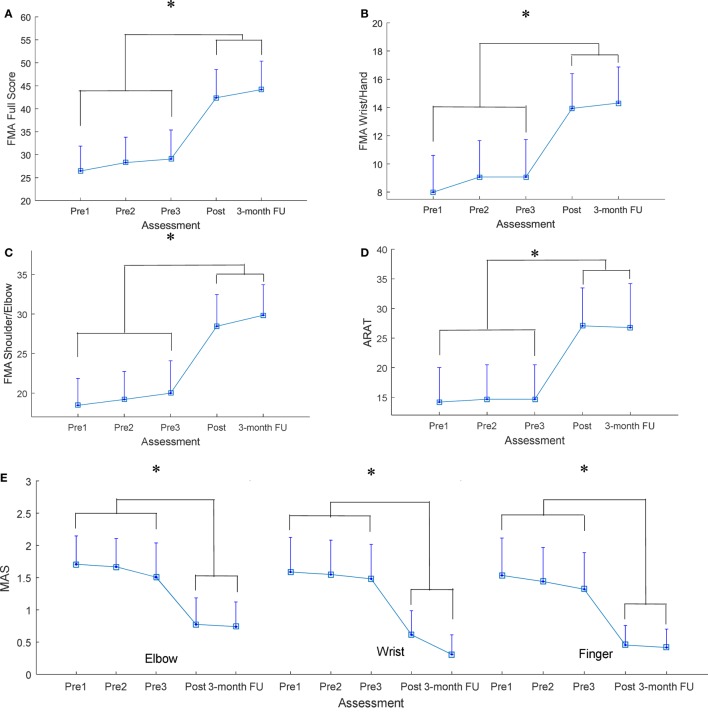
The clinical scores measured before, after, and 3 months later after the training **(A)** Fugl-Meyer Assessment (FMA) full score, **(B)** FMA wrist/hand score, **(C)** FMA shoulder/elbow score, **(D)** Action Research Arm Test (ARAT) score, **(E)** Modified Ashworth Scale (MAS) score at the elbow, the wrist, and the fingers, presented as mean value with two times SE (error bar) in each evaluation session. The significant difference is indicated by “*” (*P* < 0.05, one-way analysis of variance with Bonferroni *post hoc* tests).

All recruited participants (*n* = 15) completed the EMG-driven NMES-robotic hand-assisted upper-limb training. The demographic data of the participants are shown in Table 1. Table 2 lists all clinical scores measured in this study (i.e., the means and 95% confidence intervals of each clinical assessment together with the one-way ANOVA probabilities with the effect sizes (EFs) for the evaluation with respect to the assessment sessions). Significantly difference clinical scores (*P* < 0.05, one-way ANOVA with Bonferroni *post hoc* test) are illustrated in Figure 4, which shows the FMA, ARAT and MAS scores evaluated at five time points: thrice pretraining assessments (Pre1, Pre2, and Pre3), posttraining assessments (Post), and 3-month follow-up assessment (3-month FU). Figures 4A–C show the variation in FMA scores at thrice pretraining assessments, posttraining assessment, and 3 months follow-up assessment. In Figure 4A, the FMA full score significantly increased after the training, and this increase compared with the pretraining values was kept for 3 months (*P* < 0.001, EF = 0.313, *F* = 7.96, one-way ANOVA with Bonferroni *post hoc* test). In Figure 4B, a significant increase of the FMA wrist/hand score was detected after the training, and the increments with respect to the pretraining value were maintained 3 months (*P* < 0.001, EF = 0.228, *F* = 5.18, one-way ANOVA with Bonferroni *post hoc* test). A significant increase in the FMA shoulder/elbow score after the training was observed compared with the pretraining values, and the increase was maintained during the assessment at the 3-month follow-up (Figure 4C; *P* < 0.001, EF = 0.320, *F* = 8.23, one-way ANOVA with Bonferroni *post hoc* test). The variation in ARAT score at five time points is shown in Figure 4D. A significant increase in the ARAT score after the training was observed, and this increase compared with the pretraining values was maintained for 3 months (*P* < 0.01, EF = 0.226, *F* = 5.12, one-way ANOVA with Bonferroni *post hoc* test). Figure 4E shows the variation in MAS scores at the finger, wrist, and elbow at five time points. A significant decrease in the MAS scores was observed in the assessments at different time points. The MAS scores at the elbow significantly declined after training, and these decreases compared with the pretraining values were maintained for 3 months (*P* < 0.01, EF = 0.214, *F* = 4.77, one-way ANOVA with Bonferroni *post hoc* test). Significant decreases were observed in the MAS score at the wrist (*P* < 0.001, EF = 0.224, *F* = 5.64, one-way ANOVA with Bonferroni *post hoc* test) and finger (*P* < 0.001, EF = 0.236, *F* = 5.41, one-way ANOVA with Bonferroni *post hoc* test) after the training, and these deductions with respect to the pretraining values were maintained during the 3-month follow-up.

**Table 1:** Demographic characteristics of the stroke subjects.

Subjects no.	Gender (female/male)	Stroke Types (hemorrhagic/ischemic)	Side of hemiparesis (left/right)	Age (years), mean ± SD	Years after onset of stroke, mean ± SD
15	3/12	7/8	8/7	57.3 ± 8.87	8.26 ± 4.17

**Table 2:** The means and 95% confidence intervals for each measurement of the clinical assessments, and the probabilities with the estimated effect sizes of the statistical analyses.

Evaluation	Pre 1	Pre 2	Pre 3	Post	3-Month follow-up	One-way ANOVA	
	
	Mean (95% confidence interval)					*P*-value (partial η^2^)	*F*-value
**FMA**							
Full score	26.5 (21.1–31.9)	28.3 (22.7–33.8)	29.1 (22.7–35.4)	42.4 (36.3–48.5)	44.2 (38.0–50.3)	0.000*** (0.313)	7.96
Wrist/hand	8.0 (5.4–10.6)	9.1 (6.5–11.6)	9.1 (6.4–11.7)	13.9 (11.4–16.4)	14.3 (11.7–16.9)	0.000*** (0.228)	5.18
Shoulder/elbow	18.5 (15.1–21.9)	19.2 (15.7–22.7)	20 (15.9–24.1)	28.5 (24.5–32.5)	29.8 (26.0–33.7)	0.000*** (0.320)	8.23

ARAT	14.2 (8.4–20.0)	14.7 (8.2–20.5)	14.7 (8.8–20.5)	27.1 (20.7–33.4)	26.8 (19.4–34.2)	0.001** (0.226)	5.12

**WMFT**							
Score	40.5 (29.7–51.2)	40.9 (30.7–51.0)	39.5 (29.5–49.5)	46 (39.2–52.8)	49.3 (42.4–56.2)	0.532 (0.043)	0.79
Time	50.0 (35.8–64.2)	49.6 (35.6–63.6)	50.5 (36.0–64.9)	39.6 (30.0–49.3)	37.7 (28.2–47.2)	0.424 (0.053)	0.98

FIM	65.0 (63.8–66.1)	65.8 (65.3–66.3)	65.6 (64.7–66.5)	66.5 (65.8–67.1)	65.7 (64.7–66.7)	0.177 (0.085)	1.63

**MAS**							
Elbow	1.7 (1.3–2.1)	1.7 (1.2–2.1)	1.5 (1.0–2.0)	0.8 (0.4–1.2)	0.7 (0.4–1.1)	0.002** (0.214)	4.77
Wrist	1.6 (1.0–2.1)	1.5 (1.0–2.1)	1.5 (0.9–2.0)	0.6 (0.2–1.0)	0.3 (0.0–0.6)	0.000*** (0.224)	5.64
Finger	1.5 (1.0–2.1)	1.4 (0.9–2.0)	1.3 (0.8–1.9)	0.5 (0.1–0.8)	0.4 (0.1–0.7)	0.000*** (0.236)	5.41

Differences with statistical significance (one-way ANOVA with Bonferroni *post hoc* tests) are marked with “*” beside the *P* values. Significant levels are indicated as follows: * for ≤0.05, ** for ≤0.01, and *** for ≤0.001.

*FMA, Fugl-Meyer Assessment; ARAT, Action Research Arm Test; WMFT, Wolf Motor Function Test; FIM, Functional Independence Measurement; MAS, Modified Ashworth Score; ANOVA, analysis of variance*.

**Figure 5:**
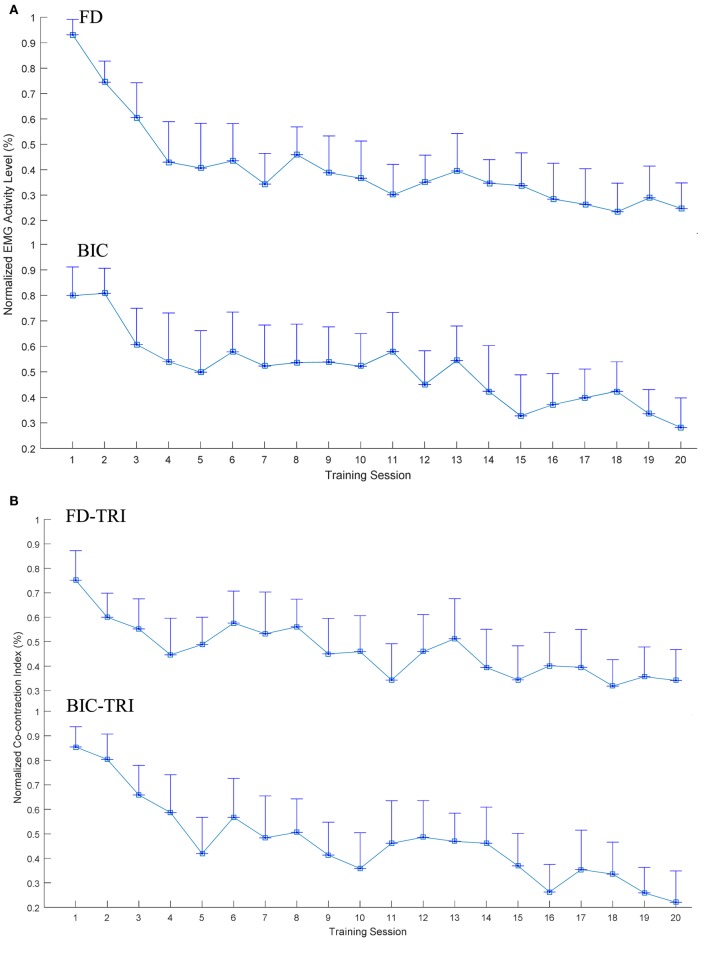
The variation of electromyography (EMG) parameters recorded across the 20 training sessions associated with significant decreases (P < 0.05 with one-way analysis of variance with Bonferroni post hoc tests): **(A)** the normalized EMG activation levels of the flexor digitorum (FD) and BIC muscles during the bare hand evaluation. **(B)** The changes of the normalized Co-contraction Indexes of the FD and TRI and BIC and TRI muscle pairs with statistical significance during the bare hand evaluation. The values are presented as mean value with two times SE (error bar) in each session.

Figure 5 illustrates the EMG parameters (i.e., the normalized EMG activation level and the normalized CI) that showed statistically significance variations during the evaluation across the 20 training sessions. A significant decrease in EMG activation level was observed in the FD (Figure 5A; *P* < 0.001, EF = 0.331, *F* = 7.29, one-way ANOVA with *post hoc* tests) and BIC muscles (*P* < 0.001, EF = 0.207, *F* = 3.85, one-way ANOVA with *post hoc* tests). Regarding the variation patterns of the EMG activation level of the FD muscle, the EMG level showed a rapid decrease of 50% over the first four sessions and was further declined by 19% from the 5th to 20th sessions. Concerning the variation patterns of the EMG activation level of the BIC muscle, the EMG level steadily decreased over the 20 training sessions, with a total decrease of 50%. No descending plateau was reached for the EMG levels of the FD and BIC muscles within the 20 training sessions. Figure 5B shows the significant decrease in CI of the FD and TRI muscles (*P* < 0.001, EF = 0.148, *F* = 2.56, one-way ANOVA with *post hoc* tests) and BIC and TRI muscle pair (*P* < 0.001, EF = 0.285, *F* = 5.88, one-way ANOVA with *post hoc* tests) during the evaluation across the 20 sessions of the training. Regarding the variation patterns of CI of the FD and TRI muscles and the BIC and TRI muscle pair, the CIs gradually declined and did not reach a plateau over the 20 training sessions. No significant increases or decreases were observed in the EMG parameters of other target muscles and muscle pairs.

## Discussion

In this study, the recruited participants with chronic stroke showed stable baselines without significant variations in all clinical scores before the training. After 20 sessions of the upper-limb training assisted with EMG-driven NMES-robotic hand, motor function improvements associated with the improved clinical scores and cross-session recorded EMG parameters were observed in all the participants, and the improvements after the training could be maintained 3 months later.

### Training Effects by Clinical Assessments

Results from the clinical assessments revealed that the voluntary motor functions and muscle coordination of the affected upper limb significantly improved after the training. The significant increase in the FMA (shoulder/elbow and wrist/hand) score after the training indicated an improvement in voluntary motor control at the joints of the entire paretic upper limb, and these motor function improvements were maintained at 3-month follow-up. A significant increase of 6 points in the FMA wrist/hand (max 24) score was observed after the training (mean admission score was 8 points). Compared with a similar study on robot-assisted hand training by using HapticKnob (10), motor improvement exhibited a significant increase of 1 point after the training (mean admission score was 8 points). Because the participants with chronic stroke in both studies practiced hand closing and opening movements through robot-assisted training, the training duration and intensity were also comparable, the additional improvements in hand functions in this study were probably due to the involvement of voluntary efforts from the affected limb and NMES during finger extension. The ARAT score is mainly related to finger movements as well as grasping, gripping, and pinching movements. The significant increase in the ARAT score indicated improvements in the muscle coordination of the fingers for fine precision grasping and joint stability of the fingers. The significant decrease in the MAS score at the elbow implied a release of flexor spasticity (muscle tone) in the elbow joint. The significant decrease in the MAS scores at the flexors of the wrist and fingers indicated that the spasticity of the distal joints was reduced. The muscle tone was graded subjectively by the examiner depending on the amount of the resistance encountered in response to passive movement (34). A higher MAS score reflects poorer control of synergic muscle activity as well as a tendency to stiffen a limb to compensate for poor control (21). Stroke survivors usually exhibit various compensatory motions while using their paretic upper limbs (30). For example, patients with stroke use trunk flexion instead of elbow extension to reach for objects. Similarly, forearm pronation and wrist flexion instead of a neutral forearm position and wrist extension to orient the hand for grasping. The decrease in the MAS scores of the elbow, wrist, and finger joints indicated improved muscle coordination and joint stability of the proximal and distal joints during arm reaching motions as well as during hand grasp and release motions after the training, and these significant improvements were maintained at 3-month follow-up. In our previous study on the EMG-driven robotic hand assisted upper-limb training of patients with chronic stroke (29), the MAS score of the finger joints decreased by a total of 0.5 points after the training with a mean admission score of 1.3 points. However, in this study, a total decrease of 1 point in the MAS score of the finger joints was demonstrated after the training with a mean admission score of 1.5 points. The additional decrease in the spasticity of the finger joints in this study may be due to the NMES assistance for finger extension during training. Further studies should be conducted to assess the effectiveness of training in poststroke rehabilitation of the upper limb assisted with the EMG-driven NMES-robotic hand by comparing the training results with the EMG-driven robotic hand assisted hand training in a randomized controlled trial.

A review on robot-assisted poststroke upper-limb rehabilitation (43) indicated that a significant improvement in the function of ADLs (i.e., FIM score) must be associated with a significant improvement in the motor function recovery (i.e., FMA score); however, no study has demonstrated significant improvement in ADL functions without motor recovery. Motor function recovery is considered a prerequisite for the ability to perform ADLs. In this study, significant motor function improvements (i.e., FMA and ARAT scores) have been observed, but the improvements in ADLs were not confirmed using the clinical outcome measures (i.e., WMFT and FIM scores). This might suggest that the motor function improvements after the training might not be transferred to the functional use of the upper limb to perform ADLs, which is a common observation in robot-assisted studies on patients with chronic stroke (44). This was probably due to the following features in persons with chronic stroke: (1) learned nonuse that could become a habit, and the limb may not be used in functional activities although the individual can move it (30) and (2) the unaffected limb attempts to execute all motor actions required for daily living (45). Further studies should be conducted on upper-limb rehabilitation of patients with subacute stroke using the assistance of the EMG-driven NMES-robotic hand, which might limit the occurrence of the learned nonuse and increase the functional use of the affected limb in ADLs. In contrast to the WMFT and FIM scores, the FMA, ARAT and MAS scores indicated that significant improvements in arm and hand functions could be maintained 3 months later after the training. This implied that upper-limb training assisted with EMG-driven NMES-robotic hand could provide motor function recovery for the proximal and distal joints of the impaired limb and support the retentive long-term upper-limb rehabilitation for patients with chronic stroke. It was also possible that the participants utilized the affected upper limb more confidently in the daily activities with the improved motor functions after the training, which led to the maintenance of the motor gain 3 months later. However, it did not lead to a significant improvements in the WMFT or FIM.

### Training Effects by Cross-Session EMG Monitoring

The cross-sessional EMG monitoring reflected the recovery progress of the muscle coordination during the training program, which also monitored individual muscle activation and coordination patterns among the contracting muscles. The significantly improved muscular coordination of the proximal and distal joints also was achieved through the EMG-driven NMES-robotic hand assisted training, as revealed by both clinical scores and the EMG parameters (i.e., the normalized EMG activation levels and the normalized CIs). The decrease in the EMG activation levels could have two major reasons: (1) the reduced spasticity, which reduced the extra muscle activities (46), and (2) the decreased overactivation of muscles during the initial period of motor learning for a skill-requiring task (47). The significant decrease in the EMG activation levels of the FD and BIC muscle reflected the reduced spasticity of the related joints, which was also manifested as the decreased MAS scores in the elbow, wrist, and finger joints. The significant decrease in the normalized EMG activation levels of the FD and BIC muscle across training sessions also reflected a reduction of excessive muscular activities in the FD and BIC muscle in the bare hand evaluation task during hand opening, hand closing, and arm reaching movements. The reduction of excessive muscle activities suggested improved muscle coordination and voluntary motor controls during arm transportation and hand grasp movements. These improvements also contributed to a significant increase in the FMA (shoulder/elbow and wrist/hand) scores after training. The EMG level of the FD muscle exhibited a rapid decrease of 50% over the first four sessions, and it further declined by 19% from the 5th to 20th sessions in contrast to the relatively gradual decrease of the EMG level of the BIC muscle across 20 training sessions, with a total decrease of 50%. These results demonstrated similar patterns in the motor recovery under EMG-driven NMES robot-assisted upper-limb training as observed in our previous study on the wrist rehabilitation (23). In that work, the EMG activation level of the main flexor in the wrist (flexor carpi radialis) decreased faster in a 20-session EMG-driven NMES robot-assisted wrist training program, in comparison with the training only assisted with the EMG-driven pure robot (without NMES). It suggested that the combined treatment of the robot and NMES could speed up the recovery process (23). In this study, NMES assistance on finger extension may have contributed to the faster release of excessive contraction of the FD muscle, thus further improving muscular coordination of the finger joints. While the results of the EMG levels of the FD muscle showed the acceleration of the recovery process, the EMG levels of the FD and BIC muscles did not reach a plateau within the 20 training sessions. In a review of motor learning studies, the researcher indicated that the learning of a skilled movement is characterized by a plateau of little or no change in performance (48). Therefore, the further improvement in the recovery of the FD and BIC muscles could be obtained by providing additional training sessions.

In addition to the EMG activation levels, the CI revealed the coactivity of a muscle pair and the recovery progress on muscular coordination. Dewald et al. indicated that discoordination among muscles is one of the major factors for motor disability after stroke and highly related to the muscle spasticity and compensatory motions in the affected limb (49). Compensatory movements from proximal joints during motions at distal joints were commonly observed in poststroke survivors, which resulted in excessive co-contractions in muscles related to both the proximal and distal joints (6, 30). In this work, the evolutionary patterns of muscular coactivity within a joint and across joints in the upper limb were investigated by CIs among the related muscles. A decrease in the CI value of a muscle pair indicated a release of the co-contraction between the two muscles, i.e., the two muscles could contract more independently in the desired task. The significantly decreased CI of the FD and TRI muscles indicated the reduction of the coactivity between the elbow joint and finger joints, which suggested the improved isolation of the distal joint movements from the proximal joint. The reduction in cross-joint muscles (i.e., FD and TRI) also indicated reduced compensation movement from co-contraction on the elbow joint during hand closing and opening motions. The significant decrease in the CI of BIC and TRI muscle pair was observed, and it indicated that the muscle coordination for achieving reaching motions through the elbow flexion and extension was promoted. However, the CI of the FD and TRI muscles and BIC and TRI muscle pair did not reach a plateau within the 20 training sessions. Further decreases in the CI value could be obtained by conducting additional training sessions.

In this work, the motor function improvement was obtained at the elbow, wrist and fingers as reflected by the clinical scores and the EMG parameters. During the training, the assistance from the EMG-driven NMES robot was incorporated in the coordinated tasks related to the arm reaching/withdrawing and hand open/close of the whole upper limb. Multi-joint coordinated upper limb practice simulating daily activities is necessary for stroke survivors to regain meaningful motor functions after training, since the task practiced would be the motor function restored, e.g., task-oriented rehabilitation (50). In the conventional physical rehabilitation on the upper limb, it was hard for a human therapist (or a stroke patient himself/herself in independent practices) to support the arm motions and manage the movements of the distal joints, e.g., finger joints, at the same time. This was one of the reasons that most of stroke survivors experienced reasonable recovery in the proximal joints, whereas little in the distal (51). In this work, the EMG-driven NMES-robot managed the finger motions while the stroke participants practicing the whole upper-limb tasks, which led to the motor improvements at both the proximal and distal joints. It was also noticed that the motor gains measured by FMA for the shoulder/elbow and wrist/hand were both around 20% immediately after the training (8-point increment at the shoulder/elbow with a full mark of 42 and 5-point increment at the wrist/hand with a full mark of 24). Besides the coordinated physical practice of the whole upper limb, another reason associated with the proximal recovery was related to the competitive interaction between the proximal and the distal joints in rehabilitation after stroke (52). Proximal joints (e.g., the shoulder/elbow) could gain more than the distal, e.g., the wrist/fingers, due to the compensatory activities from the proximal joints, which was related to the reduced inhibitory function of the ipsilesional motor cortex. Physical training at a distal joint benefited the motor function at the proximal joints was also observed in our previous robot-assisted wrist rehabilitation even with a fixed position of the elbow joint(14, 23).

## Limitations

It was understood that the combined treatment of NMES and robot could introduce additional muscle fatigue to the target muscle under stimulation, i.e., the ED muscle in this work, in a training program with multiple sessions. Accumulated fatigue in the stimulated muscle might result in an increase in the EMG amplitude of the target muscle across the sessions. Although normalized EMG signals were adopted in this work to minimize the cross-sessional difference in EMG detection, more sensitive EMG representations which are less affected by the muscle fatigue will be explored in our future study. In this work, there was no significant change observed in the ED EMG level, nor in the CIs related to the ED muscle across the training sessions. Randomized controlled trials will be conducted in future studies to compare the rehabilitation effectiveness of the EMG-driven NMES-robotic hand with other device assisted programs (e.g., EMG-driven robotic hand) and with the conventional manual treatment on the upper limb.

## Conclusion

In this study, the training effects of the poststroke upper-limb training assisted with EMG-driven NMES-robotic hand were investigated through a single-group clinical trial on patients with chronic stroke. The measured outcomes (i.e., clinical scores and EMG parameters) indicated that significant motor function improvements were achieved after the training, which included an increase in the voluntary motor effort on the entire upper limb, improved muscular coordination, and released muscle spasticity in the proximal and distal joints, and the motor improvements could be maintained till 3 months later after the training. Evidence suggests that intensive and repetitive upper-limb training with coordinated hand movements assisted by the voluntary EMG-driven NMES-robotic hand facilitates hand function recovery and improves muscular coordination in the upper limb with long sustainability in patients with chronic stroke.

## Ethics Statement

The study was carried out in accordance with the human ethic guidelines of the Human Subjects Ethics Subcommittee of Hong Kong Polytechnic University.

## Author Contributions

CN contributed in the NMES-robot hand-assisted training experiment, data collection and analysis, and manuscript drafting. WR and WL contributed in the NMES-robot design and maintenance. YX contributed in the training and data collection. XH conceived of the study and coordinated the whole project, including the trial design, human subject experiments, and manuscript drafting. YZ contributed in the data analysis and manuscript drafting.

## Conflict of Interest Statement

The authors declare that the research was conducted in the absence of any commercial or financial relationships that could be construed as a potential conflict of interest.
